# A Lethal Change in the Weather: Temperature Extremes and Premature Mortality

**Published:** 2006-09

**Authors:** Nancy Bazilchuk

Extremely hot and cold days can be fatal to certain vulnerable populations, as the more than 160 deaths in two weeks during California’s July 2006 heat wave clearly showed. The elderly and lower-income individuals are generally acknowledged to be most vulnerable to the effects of temperature extremes, but relatively little is known about how such extremes combine with underlying medical conditions to increase mortality risks. Now, a team from the Harvard School of Public Health has analyzed millions of death records from 50 U.S. cities to identify factors that increase the risk of dying on extremely hot or cold days **[*EHP* 114:1331–1336; Medina-Ramón et al.]**. The study is the first of its size to identify specific diseases that produce the largest relative mortality increases on extreme temperature days.

The researchers examined approximately 7.8 million death records for the period 1989 through 2000. They defined extreme temperatures for each city as the coldest 1% of daytime highs and the warmest 1% of nighttime lows. These are the most physically challenging conditions, with people unable to warm up even in the daytime or cool off even at night. The data were analyzed using a case-only approach, a technique borrowed from genetic research that allows the identification of time-invariant factors (such as gender) that modify the effect of a time-variant risk factor (such as weather). This approach allowed the researchers to compare the individual characteristics of those dying under extreme weather conditions with those dying on other days.

The study’s large sample size provided enough statistical power for researchers to see how a variety of individual characteristics, including presence of chronic conditions, affected vulnerability to weather extremes for a specific cause of death. For example, previous studies had already shown that blacks are more likely than whites to die on a hot day, but the authors found that susceptibility in this subgroup was more pronounced when death was due to cardiovascular disease. Conversely, the elderly and diabetics were more vulnerable to heat when the primary cause of death was *not* due to cardiovascular disease. The researchers also found a large increase in vulnerability to heat in individuals who suffered from atrial fibrillation, a finding that has not previously been reported. Cardiovascular deaths, especially cardiac arrest deaths, also showed a greater relative increase on extremely cold days.

The authors note that public health officials can target appropriate health services and infrastructure by knowing which subpopulations are particularly at risk from temperature extremes as well as the most common mortality causes that may affect them. This kind of information will be even more important as some subgroups (such as diabetics and the elderly) increase as a percentage of the population at large, and as global warming raises the probability of higher maximum temperatures.

## Figures and Tables

**Figure:**
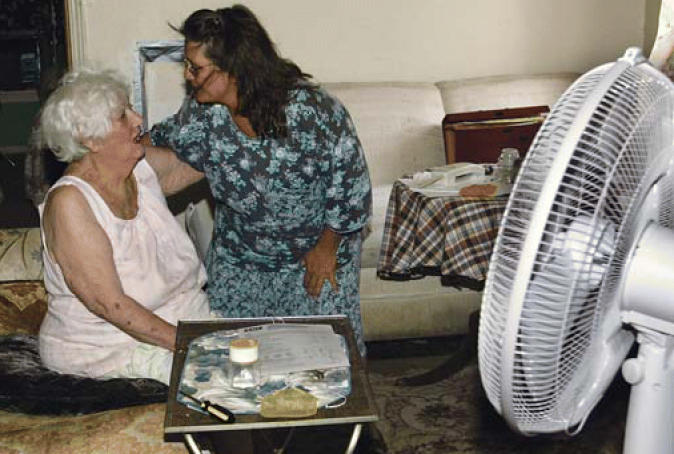
Hot science Identifying disease risks related to extreme temps can help officials target services such as fan distribution to those most at risk.

